# On the Origins of a Species: What Might Explain the Rise of *Candida auris*?

**DOI:** 10.3390/jof5030058

**Published:** 2019-07-06

**Authors:** Brendan R. Jackson, Nancy Chow, Kaitlin Forsberg, Anastasia P. Litvintseva, Shawn R. Lockhart, Rory Welsh, Snigdha Vallabhaneni, Tom Chiller

**Affiliations:** 1 Centers for Disease Control and Prevention, Division of Foodborne, Waterborne, and Environmental Diseases, Mycotic Diseases Branch, Atlanta, GA 30329, USA; 2 IHRC, Inc., Atlanta, GA 30346, USA; 3 Centers for Disease Control and Prevention, Division of Healthcare Quality Promotion, Prevention and Response Branch, Atlanta, GA 30329, USA

**Keywords:** *Candida auris*, yeast, ecological niche, fungal infection, emerging infections

## Abstract

*Candida auris* is an emerging multidrug-resistant yeast first described in 2009 that has since caused healthcare-associated outbreaks of severe human infections around the world. In some hospitals, it has become a leading cause of invasive candidiasis. *C. auris* is markedly different from most other pathogenic *Candida* species in its genetics, antifungal resistance, and ability to spread between patients. The reasons why this fungus began spreading widely in the last decade remain a mystery. We examine available data on *C. auris* and related species, including genomic epidemiology, phenotypic characteristics, and sites of detection, to put forth hypotheses on its possible origins. *C. auris* has not been detected in the natural environment; related species have been detected in in plants, insects, and aquatic environments, as well as from human body sites. It can tolerate hypersaline environments and higher temperatures than most *Candida* species. We explore hypotheses about the pre-emergence niche of *C. auris*, whether in the environmental or human microbiome, and speculate on factors that might have led to its spread, including the possible roles of healthcare, antifungal use, and environmental changes, including human activities that might have expanded its presence in the environment or caused increased human contact.

## 1. Introduction

Until a few years ago, *Candida auris*, a fungus first described in 2009 [1], was an obscure yeast unknown in most parts of the world, even among mycologists. However, by 2016, following *C. auris* outbreaks in several countries, public health agencies issued alerts to healthcare providers [2,3,4]. These alerts highlighted concerns about the challenges in correct identification, the species’ multidrug-resistance, spread in healthcare facilities, and association with high mortality. Although other *Candida* species can cause outbreaks rarely [5], *C. auris* appears to possess an unprecedented ability among known pathogenic fungi to spread between patients in healthcare facilities, likely related to its ability to colonize human skin and persist for long periods [6], survive on surfaces for several weeks [7], and tolerate some commonly used healthcare disinfectants (e.g., quaternary ammonium compounds) [8]. The reasons for the rapid emergence of this organism as a cause of human disease are unknown. This article summarizes available data to inform our understanding about the possible origins of *C. auris*. The hypotheses arising from this information remain speculative, and the purpose of this article is to stimulate research to determine origins of *C. auris,* with the aim of helping prevent future spread of this organism and other pathogens yet to emerge. The ideas presented here represent the diverse and sometimes diverging opinions of the authors, and this lack of consensus underscores how little is known about the origins of *C. auris* and the need for further study. 

## 2. First Detection and Known Global Distribution

Human cases of *C. auris* infection have been reported on every inhabited continent, but ongoing transmission so far has been limited to roughly a dozen countries [9,10]. First described in Japan in 2009 and shortly thereafter as a cause of invasive infections in South Korea in 2011 [11], *C. auris* does not appear to have taken root in these countries, causing only sporadic rather than epidemic infections [12]. In contrast, extensive transmission has been documented in South Asia (India and Pakistan), South America (Colombia and Venezuela), and South Africa [10]. Transmission has also been documented in the United States, the United Kingdom, Spain, Panama, and other countries [9,10]. In some hospitals where *C. auris* has taken hold, it has caused over 40% of *Candida* bloodstream infections after being previously absent only a year or two earlier [13]. 

A common question about *C. auris* is whether it might have been long present in the clinical setting, its emergence being an artifact of the dissemination of improved yeast identification methods, such as DNA sequencing and mass spectrometry, as *C. auris* is commonly misidentified by other techniques. Available evidence suggests that this is not the case. The SENTRY Antifungal Surveillance Program, which included >20,000 isolates collected from 150 medical centers in >40 countries during 1997–2016, did not identify *C. auris* until 2009 [14]. Similarly, sentinel candidemia surveillance at multiple U.S. sites dating back to 2008 did not detect *C. auris* before 2017 (CDC, unpublished data). At present, the earliest known retrospectively identified *C. auris* isolates date back to 1996 in South Korea, 1997 in Japan [10], and 2008 in Pakistan; however, earlier unidentified cases almost certainly occurred, and further examination of historic isolate collections for *C. auris* may help shed light into its origins as a human pathogen.

## 3. Genomic Epidemiology 

Whole-genome sequencing (WGS) of 54 *C. auris* isolates from four world regions (East Asia, South Asia, Africa, and South America) revealed four genetically distinct populations or major clades of *C. auris,* a puzzling finding that supports independent and nearly simultaneous emergence in geographically separated human populations. Specifically, all isolates within each region (albeit limited to one to three countries each) have been highly related—nearly clonal—but isolates have been highly distinct (tens of thousands of single nucleotide polymorphisms, SNPs) across regions [14]. Consistent with this geographic clonal distribution, all isolates from cases identified in the United States, which have been tracked intensively since 2016, have been highly related by WGS to isolates from each of these 4 regions. In several cases, patients had recently been hospitalized in a country where highly related isolates were detected [15,16]. WGS of a recently identified isolate from Iran suggests a fifth major clade of *C. auris* may exist, with hundreds of thousands of SNPs separating this isolate from the four known clades [17]. Continued surveillance and characterization of *C. auris* isolates may confirm the existence of a fifth *C. auris* clade. It is with trepidation that we refer to the four clades by geographic region, as the detection of phylogeographically distinct strains might be explained not only by emergence from local sources in each of those four regions, but might also arise from the founder effect and subsequent local spread in an era of extensive global travel. It is possible that a strain detected in one region might have originated in another. For example, had *C. auris* never been detected in South and Central America, we might suspect that the U.S. state of Illinois, where closely related isolates are being transmitted [16], was the initial source of this strain. It is even possible that all four clades—and perhaps others yet to be confirmed—could have coexisted in a single geographical region until global travel or trade introduced one clade each into human populations in the four regions in which the clades have been found, followed by local clonal spread (i.e., founder effect). Additionally, gaps in detection capacity in many countries, particularly in those in Africa, Asia, and Latin America, make it likely that *C. auris* exists in other places undetected. Improved laboratory infrastructure and WGS analysis of additional isolates, particularly from outside the United States and of existing historical collections, may help shed light on these questions. 

Another question arising from the genomic epidemiology of *C. auris* is why the East Asian clade has not been implicated in outbreaks, in contrast to the other three clades, despite having been isolated from persons in multiple countries for the largest number of years (i.e., since 1996) [11,12,16]. Although epidemiologic and contextual (e.g., infection control and other health system differences) reasons may explain this finding, it is possible that this clade is substantially different from the others in its transmissibility and possibly its environmental source. Supporting this hypothesis, most *C. auris* external otitis or ear colonization have been caused by strains in the East Asian clade [18], whereas invasive infections with this clade have been less common. 

Regardless, the existence of multiple distinct *C. auris* strains suggests that it has been on Earth for many years given the amount of time needed to produce its levels of genetic diversity. Why then have three clades of *C. auris* begun emerging now, causing transmissible and invasive infections on a large scale only in the past decade in middle- and high-income countries with detection capabilities? The biological properties of *C. auris* and information about related species may provide clues to its origins.

## 4. Distinctive Biological Properties of *C. auris*

*C. auris* shares the genus *Candida* with common human commensals like *C. albicans* and *C. glabrata*—the *Candida* species that cause most human infections in North America and Europe—which might suggest that it is closely related to these organisms. However, *Candida* is a broad and polyphyletic genus with over 500 species, many markedly different from one another. *C. albicans* is reportedly as genetically divergent from *C. glabrata* as humans are from fish [19]. Although *C. auris* belongs to the same CTG clade as *C. albicans*, *C. parapsilosis*, and other *Candida* species that translate the CTG codon as serine instead of leucine, considerable genetic distance exists between *C. auris* and most other pathogenic *Candida* species [20], which are classified in the Debaryomycetacae family [21]. *C. auris*, on the other hand, has been classified within the *Clavispora* clade of the Metschnikowiaceae family [21], a group of yeasts isolated principally from non-human sources. *Candida lusitaniae*, the fifth or sixth most frequent cause of candidemia in the United States (and sometimes resistant to multiple antifungal classes) [22,23], is also classified in the Metschnikowiaceae family [21,24].

*C. auris* causes invasive infections similar to those caused by *C. albicans* and *C. glabrata*. However, the long absence of *C. auris* in SENTRY data might suggest that *C. auris* has not long been a colonizer of humans. On the other hand, SENTRY and most isolate collections focus on invasive isolates and may not have detected *C. auris* if colonization had not previously led to invasive infection. For example, a recent study of the human skin mycobiome identified numerous fungal species, some previously undescribed, that have never been known to cause infection [25]. However, a sizeable proportion (4%) of colonized patients in some healthcare facilities have gone on to develop *C. auris* bloodstream infections [26]. If *C. auris* has long been part of the human microbiome, it must have recently acquired sufficient virulence to cause invasive infections or been newly introduced into human populations in which invasive *Candida* infections can be identified. That *C. auris* readily colonizes patients’ skin and other body sites (e.g., nose, ears, mouth, rectum, vagina) [6,7,15,27,28] raises the question of why it did not spread previously if it had long been part of the human microbiome. 

*C. auris* possesses distinctive properties compared with many other *Candida* species, including its close relatives. It can grow at temperatures as high as 42 °C (108 °F) [24,29,30]. Although many people associate fungal growth with warm conditions, few fungi can grow at temperatures at or above 37 °C. In one study, the number of surviving fungal isolates declined by 6% for every 1 °C increase in the 30–40 °C range [31]. The thermotolerance of *C. auris* allows it to cause invasive human infections, including tolerating the fever response, and has led to speculation that it may be able to infect birds [24], whose body temperatures usually range 40–42 °C, raising the possibility of an avian reservoir. Second, *C. auris* tolerates hypersaline conditions several times saltier than the ocean. A *C. auris* enrichment broth containing 10% NaCl incubated at 40 °C inhibited the growth of most other yeasts, including many in the closely related *Candida haemulonii* complex [7]. This thermotolerance and halotolerance might allow *C. auris* to survive environments like hypersaline desert lakes, salt-evaporating ponds, or tidal pools. Although these locations may not be the natural habitat of *C. auris*, its ability to survive on dry environmental surfaces for weeks suggests it is well-adapted to survival outside host organisms. Thermotolerance and halotolerance would also be advantageous for an organism that needed to survive on skin, especially in the axilla and groin, the most common sites of *C. auris* isolation, which are prone to high temperatures and high salinity during periods of intense physical activity. 

Research to date has found that *C. auris* likely possesses some of the same virulence factors as *C. albicans*, although much remains to be characterized [20]. Co-evolution with humans is likely not a requirement for invasive infections, though it may facilitate the transition from a commensal to an “accidental” pathogen. Accordingly, it has been asserted that “virulence in opportunistic pathogens should be regarded as an evolutionary accident, rather than an evolutionary goal in itself” [19]. On the other hand, the superior ability of *C. auris* to colonize human skin successfully competing with the skin microbiome suggests that this organism might have co-evolved with the warm-bloodied animals with similar features, such as humans, other mammals, or birds. 

## 5. Taxonomy and Phylogeny of *C. auris* and Close Relatives

We may be able to infer something of the origins of *C. auris* by examining its relatives. No reports exist to date of isolation of *C. auris* from the natural environment or from animals. A recent publication reported isolation of *C. auris* from swimming pools in the Netherlands [32], a puzzling finding that might suggest that *C. auris* is well suited to aquatic environments or that it is shed by human hosts, but further evaluation of these data is needed. Yeasts in the natural environment of North America and Europe have been characterized to some degree, but sampling for yeasts in other areas, particularly tropical regions, has been limited [33]. When sampling does occur, new species are frequently detected. In addition, we have only begun to understand the human mycobiome, and studies continue to identify previously uncharacterized fungi [25,34,35]. Millions of fungal species are estimated to exist, yet only about 100,000 have been described [36]. Given the haphazard nature of sampling, when making inferences about the possible origins of *C. auris* by examining closely related species, we must acknowledge that the location in which an organism was detected does not necessarily fix its niche; the fungus or related species may inhabit starkly different habitats. 

We will first examine species related to *C. auris* that have been classified in the *C. haemulonii* complex, which includes *C. haemulonii*, *C. duobushaemulonii*, and *C. pseudohaemulonii* [37]. Like *C. auris*, these species are commonly resistant to multiple antifungal classes [38,39], an area that also warrants further exploration. To date, comparative genomic analysis of *C. auris* and related species has identified genetic features unique to this multidrug-resistant clade, including expanding families of transporters and lipases, as well as mutations and copy number variations in genes linked to antifungal resistance in other *Candida* [20]. We will then examine other named species closely related to *C. auris* and this complex, including those with temporary names (Appendix A). Note that some of the following species names and designations will likely change with time; the purpose of this list is not to classify these organisms but rather to summarize where yeasts closely related to *C. auris* have been found. As an example of the complexities in taxonomy, MycoBank lists as legitimate the names *C. haemuloni* and *C. haemulonis* instead of *C. haemulonii* and *C. duobushaemulonis* instead of *C. duobushaemulonii* [40]; here, we use *C. haemulonii* and *C. duobushaemulonii*, as most literature to date uses those names. 

*C. haemulonii*

- First described in 1961 as *Torulopsis haemulonii* after having been isolated from the gut of the blue-striped grunt fish (*Haemulon sciurus*) near the U.S. Florida coast [41].
- Other marine settings:
  ∘ Seawater near Portugal in 1962 [41] and India in 2011 [42]
  ∘ Soft coral (*Palythoa variabilis*) in Brazil in 2016 [43]
  ∘ Skin of a dolphin (species not reported) near Suriname [44]
  ∘ Second most common yeast from pool water of captive bottlenose dolphins (*Tursiops truncates*) in the U.S. state of Connecticut [45]
  ∘ Indian researchers applied *C. haemulonii* to giant tiger shrimp (*Penaeus monodon*)—widely farmed in aquaculture—finding that its presence boosted an immunostimulatory molecule in the shrimp, conferring protection against a viral infection that causes economic losses from shrimp die-off [42]. It is unclear whether *C. haemulonii* has become used more broadly in aquaculture since publication of the study in 2011. Further investigation of its use and of whether the isolate used was truly *C. haemulonii* would be of great interest.
- Terrestrial sources:
  ∘ From roots of cassava (*Manihot esculenta* Crantz) in Brazil in 2010 [46]
  ∘ From a laboratory tick colony (*Ornithodoros moubata*), where it was reportedly causing an epidemic of unexplained tick deaths in the Czech Republic in 2001 [47].
  ∘ Indian researchers applied what they reported as *C. haemulonii* to waste from dairy production [48]; however, in 2008, this strain (MTCC 1966) was determined to be *C. metapsilosis* and not in fact *C. haemulonii* [49].
- Human sources: isolated numerous times from humans, including both incidental isolation and in association with infections, mostly wound and other superficial infections but also candidemia [37,39,50,51,52,53].
- It is important to note that reports of *C. haemulonii* without use of ribosomal DNA sequencing, and before naming of *C. auris* in 2009, must be examined with caution, since misclassification as other yeasts is possible.

*C. duobushaemulonii*

- Isolated from patients’ blood and foot ulcers in Asia, Europe, and North America [37,38,39,52,53] (CDC, unpublished data).
- A cause of recurrent vulvovaginal candidiasis in Brazil [54].
- Isolated from a firebug (*Pyrrhocoris apterus*), in Germany [37].
- No other published reports exist of its isolation from the natural environment.

*C. pseudohaemulonii*

- Isolated from blood of patients in Thailand and South Korea [37].
- Yeast tentatively identified as *C. pseudohaeumulonii* isolated from a human nail in Argentina [55].
- No published reports of isolation from the natural environment.

*Candida heveicola*

- Isolated from sap from a rubber tree (*Hevea brasiliensis*) in Yunnan Province, China, in 2008 [56].

*Candida ruelliae*

- Isolated from *Ruellia* sp. flowers in India in 2008 [49].

*Candida vulturna*

**Figure 1:**
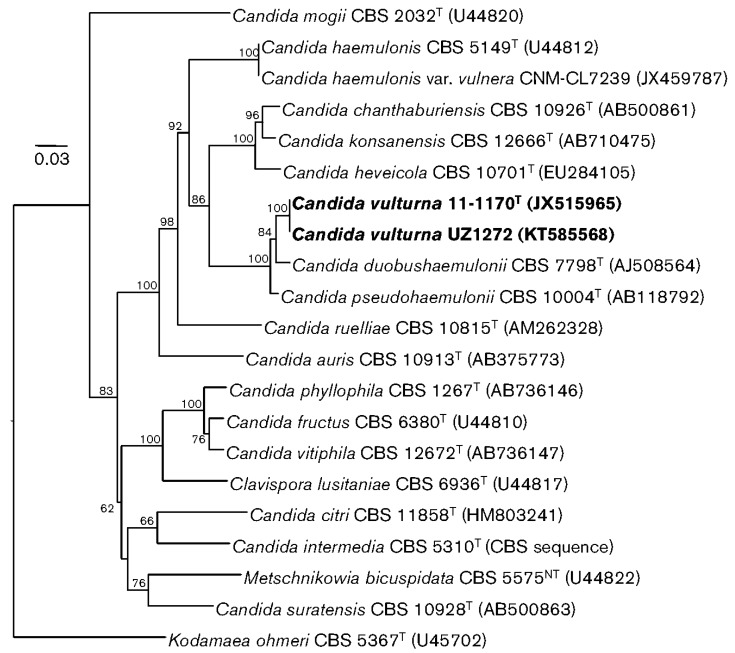
Phylogenetic relationships of *Candida auris* with *Candida vulturna* strains (in bold from original publication) and other related species and genera determined from a neighbor-joining analysis of sequences of the D1/D2 domains of the LSU rRNA genes. The sequence of the type strain of *Kodamaea ohmeri* was the outgroup in the analysis. Bootstrap values are given at branch nodes. Bar, 0.03 changes per position. Reproduced with permission from Sipiczki M and Tap RM, *Candida vulturna* pro tempore sp. nov., a dimorphic yeast species related to the *Candida haemulonis* species complex isolated from flowers and clinical sample; published by Microbiology Society, 2016 [57].

- Isolated from blood of a patient who died of aspiration pneumonia in Malaysia [57].
- Twice identified from human sources (CDC, unpublished data)
- Isolated from an unspecified flower collected on the outskirts of the city of Cagayan de Oro, Philippines, in 2016 [57]. The authors report, “as most of the other related species were originally described from plants, we presume that the natural habitat of *C. vulturna* is associated with plants but, occasionally, it can also infect humans” [57]. This report contains the most comprehensive phylogenic tree published to date about the species most closely related to *C. auris* by ribosomal DNA sequencing (Figure 1).

*Candida chanthaburiensis*

- Isolated from bark of tall-stilt mangrove (*Rhizophora apiculata*) in Thailand in 2004. The authors report that a wide variety of yeasts can be found in mangrove forests, which provide large quantities of organic matter [58].

*Candida konsanensis*

- Isolated from bluegrape jasmine (*Jasminum adenophyllum*) in Thailand [59].

In summary, known yeasts most similar to *C. auris* by DNA sequencing have been isolated from plant and marine settings and human infections. *C. haemulonii* and *C. duobushaemulonii* have been more commonly isolated from humans than non-human sources, which might indicate a human reservoir; however, testing of human isolates is likely more common than testing of environmental isolates. Apart from the yeasts described above, most other yeasts in the Metschnikowiacae family are classified not in the genus *Candida* but predominantly in the genus *Metschnikowia*. Similar to the yeasts described above, *Metschnikowia* species are also commonly associated with plants, particularly nectar and fruit, as well the insect pollinators and fruit-feeders that visit them. However, one *Metschnikowia* clade has a strong association with marine animals [60], and two isolates have been identified from human bloodstream infections (CDC, unpublished data). The fact that many isolations of these organisms have occurred in tropical areas, predominantly in South and Southeast Asia, raises the question of whether this area fostered the evolutionary origin of the *Metschnikowia* clade, particularly since fungal diversity is estimated to be greater in tropical rather than temperate regions [61] and because yeasts in the North American and European environments have been better characterized [33]. Tempering this speculation, species of *Candida* commonly associated with the human microbiome and infection are occasionally isolated from the environment. For example, a wide variety of *C. albicans* strains were recently isolated from oak trees in the United Kingdom, calling into question whether this species is truly an obligate human commensal [62]. The authors suggest that the paucity of environmental *C. albicans* isolates has more to do with lack of sampling than its abundance in the environment. Similarly, *C. tropicalis*, *C. parapsilosis*, and *C. glabrata* have been isolated from trees in Canada, and the strains found in humans differed substantially from those found on trees [63]. Recent genomic studies have also called into question whether *C. glabrata* is a human commensal, with some authors speculating that it is a generalist within the environment [64]. Similar to *C. auris*, *C. glabrata* isolates comprise different clades with evidence of geographic specificity. 

It is important then for those of us focused on human health to recognize that the natural environment contains an abundance of diverse yeasts, often adapted to harsh environments, and that local and global environmental changes can result in changes in the distribution of yeasts and how humans interact with them. As one example, the surface of plants, known as the phylloplane, contains a diverse array of yeasts, varying substantially by location. The size of this habitat is immense, with leaf surface of tropical rainforests alone estimated at 140 million km^2^ [33]. Yeasts adapted to the phylloplane must tolerate often harsh conditions involving limited nutrient availability, frequent dry conditions, and solar radiation [65]. For this reason, many yeasts have characteristics that may be useful in biotechnology applications [66], as seen with *C. haemulonii* and shrimp aquaculture [42], and these uses may lead to alterations in their environmental distribution. In an example of the interconnectedness across microenvironments, plant-associated yeasts often colonize insects that feed on plants and aid in yeast dispersal [66]. Wasps have been shown to harbor *Saccharomyces cerevisiae* over winter, providing a key environmental niche for this fungus [67]. This interconnection may explain why relatives of *C. auris* have been found on insects. 

## 6. Why Emerge Now?

Multiple phylogenetically distinct clades of *C. auris* have emerged nearly simultaneously as causes of human infections, but why have they emerged now? Possible factors include:

### 6.1. Role of Healthcare

In the United States, evidence is strong in supporting *C. auris* transmission within and across healthcare networks [16], particularly within high-acuity long-term care facilities [68], similar to the spread of bacterial multidrug-resistant organisms [69]. Similar healthcare amplification has been observed in other countries [6,27,70,71,72], whereas little evidence exists for community transmission. However, because *C. auris* is an opportunistic organism, community transmission may go undetected since most community colonized persons would not be at risk for invasive infection. Still, healthcare environments, particularly those with gaps in infection control and that provide long-term high-level care to severely ill or disabled patients, seem particularly suited for promoting transmission. Likely reasons include:

- The ability of *C. auris* to persistently and asymptomatically colonize human skin.
- The ability of *C. auris* to persistently contaminate indoor surfaces, combined with tolerance of some commonly used healthcare disinfectants.
- Concentrations of people with decreased immunity, invasive devices, and disrupted microbiomes, including from broad-spectrum antibacterial medications, antifungal medications, and underlying illness. Differences in bathing and skin care practices between the general population and those in long-term medical care, including the use of antiseptics, might also affect the skin microbiome.

There seems to be no question regarding whether healthcare has promoted the spread of *C. auris*, but a question remains: why now? Increasing access to such medical care, including increasing concentration of ill people in healthcare facilities that may have suboptimal infection control likely created additional opportunities for the spread of *C. auris* into healthcare settings. But the speed at which multiple clades of *C. auris* began spreading in healthcare facilities around the world raises the question of whether other changes, either in the organism or in the broader environment, might have promoted introductions into healthcare. 

### 6.2. Possible Changes in the Organism

Much remains unknown about the basic biology of *C. auris*, although this knowledge is rapidly expanding [20]. Future studies may shed light on whether recombination, sexual or otherwise, or other biological changes have recently increased the organism’s transmissibility or virulence. *C. auris* shares virulence and drug-resistance characteristics with species in the *C. haemulonii* clade [20], which suggest that many of these characteristics are not newly acquired. Further, since at least three clades (South Asian, South American, and African) have caused clearly documented epidemics of human infections, it seems unlikely that all would have acquired such capacity only recently unless subject to new and intense selection pressure or if they are more closely related than SNP analysis would suggest, perhaps because of sexual recombination. If very recent genomic changes are largely responsible for the emergence of *C. auris*, which seems unlikely given knowledge to date, more study will be needed to understand what selection pressures occurred and to predict what other fungi might acquire such pathogenic characteristics.

The East Asian clade of *C. auris* has been primarily associated with ear colonization and not with infection [73]. Compared with the other three clades, which involve nearly clonal isolates, isolates from the East Asian clade exhibit higher genetic diversity, which is often an indication of an older natural population that could be the progenitor of *C. auris*. It is known that that there is a genetic determination for the composition of cerumen (ear wax), and that cerumen comes in at least two forms, typically dry in Asian populations and wet in African and European populations, which might allow independent evolution of a yeast species occupying that niche [74]. It is also known that earwax contains high concentrations of oligopetides, many of which possess antimicrobial properties, as well as lipids [75], which may be relevant given that recent comparative genomic analysis indicate that gene families encoding oligopeptide transporters and lipases are expanded in *C. auris* and related species compared with other *Candida* species [20]. The fact that the three other clades colonize the skin of people in healthcare facilities so well might support an origin of coevolution with humans rather than three independent introductions from environmental niches. If human bodies have long been the reservoir of *C. auris*, however, the fact that it only recently began causing wide-scale human infections is puzzling.

### 6.3. New Human Activities—Increased Contact

Another possible explanation for the emergence of *C. auris* is that this fungus has long possessed the characteristics that make it a dangerous opportunistic pathogen but that humans had little contact with it until recently—or at least insufficient contact to introduce it into high-acuity medical settings. The ecological niche of *C. auris* remains unknown, and it is possible that humans have intruded more heavily into the plant, insect, soil, or aquatic habitat of *C. auris*, allowing for greater human colonization. Examples of such intrusive activities include deforestation, expansion of farmland, and coastal ecosystem disruption [76]. These activities likely place growing numbers of humans into contact with fungi that inhabit specific biogeographic niches, particularly in biodiverse tropical areas. For example, if *C. auris* had existed primarily in association with specific plants and insects in an area previously unaffected by large-scale human activity, increased human presence in the area could greatly increase chances of people becoming colonized with this fungus long enough to introduce it into healthcare settings.

An alternate explanation is that *C. auris* might have long been primarily a colonizer of certain human populations not in contact with intensive healthcare or outside groups and that increased contact with the broader human population through increased travel might have allowed introduction into healthcare settings and vulnerable populations. Likewise, it is also possible that *C. auris* has been a minor commensal in the ear canal or in some other human organ with a poorly characterized mycobiome. Recent changes in clinical practices, such as increased use of antibiotics or antifungals, could have caused a genetic change in this fungus or altered the composition of the skin microbiome in a way that allowed *C. auris* to escape its specialized niche, colonize larger areas of skin, and cause infections. 

Regardless of whether *C. auris* was only recently introduced from the environment or was a long-term colonizer, we have clear evidence of how travel and medical care have been key factors in disseminating this pathogen in the United States and other countries. 

### 6.4. New Human Activities—C. auris Amplification

Large-scale environmental changes might contribute not only to increased human contact with the ecological niches in which *C. auris* inhabit but might serve to amplify this fungus in the environment. Given that related *Candida* species have been found in association with plants (e.g., rubber tree, cassava), expansion of industrial farming is one possible example of this process. It is plausible that farming of a particular crop in an area containing a *C. auris* ecological niche might lead to amplification if the crop or its debris provided a particularly suitable habitat for this fungus. A possible contributing factor could be the use of fungicides, which by reducing the fungal diversity in fields or in runoff, might select for resistant species like *C. auris*. Specifically, triazole fungicides, some of which share similar structural characteristics with triazole antifungal medications, have been increasingly used in crop agriculture in recent decades [77,78], and use of these fungicides has been linked with triazole resistance in the fungus *Aspergillus fumigatus* [79]. Most strains of *C. auris* and other species in the *C. haemulonii* complex tested to date are resistant to one or more triazole medications (e.g., fluconazole), which might permit their survival in the presence of triazole fungicides [14]. 

Effluent from pharmaceutical manufacturing of fluconazole and other triazoles might also play a role in selecting for *C. auris* and other drug-resistant fungi. For example, in a study linking pharmaceutical effluent to drug-resistant bacteria, environmental sampling in Hyderabad, India, near pharmaceutical plants detected fluconazole at numerous sites, including in a sewage sample at a concentration >240 mg/L (or over 20-times the target serum concentration in humans) [80].

Triazoles are also used extensively on humans, both topically and systemically, and they are found on food and in preservatives for woods and plastics. It is possible that some humans reached a population threshold of azole exposure that allowed *C. auris* to emerge in competition among human flora, especially with the disruption of the natural skin microbiome through the use of antibiotics and skin disinfectants. However, whether selective pressure from environmental or medical triazoles may have contributed to the emergence of *C. auris* in the environment or on humans is unknown. It is also notable that some populations of *C. auris*, specifically in East Asia and Colombia, remain susceptible to triazoles and do not contain the point mutations for resistance seen in other populations [6]. 

If the primary environmental niche of *C. auris* is marine, coastal disruption could play a role, whether from growing land-based agriculture (e.g., rice, palm oil), aquaculture, coastal urbanization, or other reasons [81]. One of the most dramatic global environmental changes in the past few decades is the rapid expansion of shrimp aquaculture [76], particularly in Asia, which has grown nearly exponentially since the 1970s [82]. As part of this rapid growth, shrimp aquaculture has suffered from repeated bacterial and viral epidemics, causing substantial economic losses. Many aquaculturalists have been adding large amounts of antibacterial drugs to the water to prevent bacterial diseases, thus altering the microbial biodiversity of these aquatic environments [83]. Declines in bacteria often lead to fungal overgrowth. Some aquaculturalists also add yeast probiotics [84] (e.g., the fungus reported to be *C. haemulonii*, described above) in an attempt to stabilize the microbiome of their ponds. Such disrupted environments might allow for amplification of previously rare fungi, such as *C. auris*, if it were indeed a commonly water-dwelling organism. Notably, this hypothesis does not imply that *C. auris* would need to colonize the shrimp directly but rather that large-scale environmental changes could influence its abundance. As the natural habitat of *C. auris* remains unknown, it is impossible to assess at this point whether aquaculture could have played a role in its emergence. It should be noted that some of this paper’s authors find it difficult to imagine how the remarkable abilities of *C. auris* to colonize human skin and survive for weeks on the dry surfaces could have evolved in the aquatic environment.

### 6.5. Global Temperature Changes

Given that relatively few fungi can grow at human body temperature, especially at fever temperatures [31], it has been posited that as the mean global temperature increases and the gap between the ambient environmental temperature and mammalian body temperature narrows, new invasive fungal pathogens will emerge [85]. Changes in climate conditions could change the environmental distribution of fungi [61], and rising temperatures have already influenced infectious disease ecology [86]. As *C. auris* is more thermotolerant than many fungi, rising temperatures might have played a role in its emergence, but given an absence of knowledge about its natural habitat, it is impossible at this time to determine whether climatic changes played a role in its recent emergence as a human pathogen. 

Although the rapid global expansion of a novel fungal pathogen might seem surprising, such emergence has been seen widely in amphibians and bats. In the last 20 years, amphibian populations around the world have been decimated by the fungus *Batrachochytrium dendrobatidis*, the primary cause of chytridiomycosis, in what has been called the greatest disease-driven loss of biodiversity ever documented [87]. Recent genome sequencing analysis suggests that this fungus was endemic to East Asia and spread around the world through commercial amphibian trade, devastating naïve amphibian populations [88]. A different fungal disease, white-nose syndrome, caused by *Pseudogymnoascus destructans*, has killed over 5 million North American bats in the last decade, bringing some species to the brink of extinction, following likely introduction from northern Europe [89]. These two diseases provide examples of how a newly-introduced pathogenic fungus can spread rapidly following cross-continent introduction. 

## 7. Solving the Mystery Surrounding the Origins and Evolutionary Reservoirs of *C. auris*

Identifying evolutionary reservoirs for emerging pathogens, such as *C. auris*, can allow for mitigation of human activities that lead to disease and inform prevention and control strategies. The original reservoir of *C. auris* remains unknown, but the answers to this mystery might already exist in a major international sequencing repository.

Over the past decade, massive collaborative efforts to explore and characterize microbial diversity in humans, animals, and terrestrial and aquatic systems around the globe have greatly advanced our understanding of life on a planetary scale (e.g., Earth Microbiome Project, Global Ocean Sampling expedition, Pacific Ocean Virome project). However, the economic and technological barriers to accessing and analyzing these massive datasets have been a barrier to investigation. Recent advances in computing and sophisticated data discovery tools such as MetaSeek and Redbiom now allow access to thousands of sequencing samples from several major repositories: DNA DataBank of Japan, the European Nucleotide Archive, and GenBank at the U.S. National Center for Biotechnology Information (NCBI). In addition, the National Institutes of Health recently launched the STRIDES (Science and Technology Research Infrastructure for Discovery, Experimentation, and Sustainability) Initiative to reduce the technological barriers of accessing and computing on sequence datasets by moving the NCBI sequence repository data to the cloud, which has the potential to advance health and reduce the burden of disease. 

A systematic sequence investigation characterizing the global yeast diversity in the highlighted agriculture, aquatic, terrestrial, animal, and human settings where related *Candida* spp. have been detected is now possible using publicly available sequence archives. Detection of positive sequences could provide an important first step in mapping possible habitats of *C. auris* and related pathogens and direct future studies to detect *C. auris* in the natural environment. Whether these datasets yield traces of *C. auris*, we encourage scientists from a range of disciplines to engage in the search for the evolutionary reservoir of *C. auris*.

## 8. Response to *C. auris*

A global response is needed to contain the spread of *C. auris* infections, particularly in hospitals and nursing homes. Fortunately, key components to its control—hand hygiene, contact precautions, and environmental cleaning and disinfection—are also effective in controlling spread of other healthcare-associated pathogens. We urgently need better data on effective disinfection methods, interventions to reduce patient colonization, rapid tests for detecting patient colonization (to target infection control measures), and broader detection capacity across the globe in clinical laboratories [90]. To date, nearly all reported colonization has been among patients in healthcare facilities, and a better understanding is needed of the prevalence and duration of colonization in the broader population and of community transmission.

We also need to think bigger and further upstream to understand the possible evolutionary sources of *C. auris*. Identifying sources and possible amplifying factors may help us prevent future introductions of *C. auris* and new fungal pathogens into human populations, depending on what the sources and factors are. At the least, it can help direct environmental and clinical monitoring and human and animal disease surveillance to allow for more rapid detection of emerging threats. Essential to this effort is a better understanding of the ecology and distribution of yeasts and other fungi across the globe, particularly given rapidly changing environmental conditions.

## Appendix A

**Table A1:** Sites of isolation of *Candida* species closely related to *Candida auris* by ribosomal DNA sequencing analysis, grouped by host or environment type.

*Candida* species ^1^	Human	Animal	Plant	Aquatic Environment
*Candida haemulonii*	Numerous isolates from invasive and non-invasive sites ^2^	*Haemulon scirus* (blue-striped grunt) off coast of Florida, USA; Laboratory tick colony in Czech Republic; Soft coral in Brazil; Applied to farmed shrimp in India	Cassava root in Brazil	Seawater near Portugal and India; pool water of captive bottlenose dolphins
*Candida duobushaemulonii*	Blood and foot ulcers in Spain and USA; vaginal samples in Brazil	*Pyrrhocoris apterus* (firebug) in Germany	Not reported	Not reported
*Candida pseudohaemulonii*	Blood in Thailand and South Korea; tentative identification from nail in Argentina	Not reported	Not reported	Not reported
*Candida heveicola*	Not reported	Not reported	*Hevea brasiliensis* (rubber tree) sap in China	Not reported
*Candida ruelliae*	Not reported	Not reported	*Ruellia* sp. flowers in India	Not reported
*Candida vulturna*	Blood in Malaysia; two human sources in USA	Not reported	Flower in Philippines	Not reported
*Candida chanthaburiensis*	Not reported	Not reported	Bark of *Rhizophora apiculata* (tall-stilt mangrove) in Thailand	Not reported
*Candida konsanensis*	Not reported	Not reported	*Jasminum adenophyllum* (bluegrape jasmine) in Thailand	Not reported

^1^ This list of species closely related to *C. auris* is based on a review of published literature and is likely not comprehensive. Taxonomy of these species is not fully established. Relationships of *C. auris* to other species warrant further investigation through whole-genome sequencing analysis. Further environmental sampling for and testing of yeasts in the Metschnikowiaceae family would also inform understanding of *C. auris* phylogenetics. ^2^ Yeasts reported to be *C. haemulonii* have been isolated from a substantial number of human samples in different countries. Given this wide distribution, this table is not intended to be a comprehensive summary of *C. haemulonii* isolated from humans.
